# Adherence to Post-polypectomy Surveillance Guidelines at a Large District General Hospital

**DOI:** 10.7759/cureus.35516

**Published:** 2023-02-27

**Authors:** Daniel M Butler, Aye Myintmo, Harriet Flashman, Obioha C Ukoumunne, Rob Bethune

**Affiliations:** 1 Colorectal Surgery, Royal Devon University Healthcare NHS Foundation Trust, Exeter, GBR; 2 Faculty of Health and Life Sciences, University of Exeter Medical School, Exeter, GBR

**Keywords:** colonoscopy surveillance, cost saving, physician guideline adherence, colon cancer prevention, colorectal polyp

## Abstract

Introduction

In 2020, the British Society of Gastroenterologists (BSG), the Association of Coloproctology of Great Britain and Ireland (ACPGBI), and Public Health England (PHE) published joint guidelines regarding post-polypectomy surveillance. This study aimed to establish clinician adherence at the Royal Devon University Healthcare NHS Foundation Trust to the 2020 guidelines compared to the now-retired 2010 guidelines.

Materials and Methods

Data on 152 patients treated under the 2010 guidelines and 133 patients treated under the 2020 guidelines were collected retrospectively from the hospital’s colonoscopy database. Data were analysed to determine whether patients who had a colonoscopy fulfilled BSG/ACPGBI/PHE guidelines for follow-up. Costs were estimated using the price of colonoscopy in the NHS National Schedule.

Results

Approximately 41.4% (63/152) of patients were adherent to the 2010 guidelines while 66.2% (88/133) of patients were adherent to the 2020 guidelines. The difference in adherence rate was 24.7% (95% confidence interval 13.5% - 35.9%, p<0.0001).

Nearly 37% (35/95) of patients who would have been followed up under the 2010 guidelines did not receive any follow-up as a result of the 2020 guidelines. This represents a cost saving of £36,892.28 per year in our hospital. Approximately 47% (28/60) of patients treated under the 2020 guidelines had surveillance colonoscopy planned when the guidelines recommended no follow-up. If every clinician were fully adherent to the 2020 guidelines, then a further £29,513.82 per year would have been saved.

Discussion and Conclusion

Following the introduction of the 2020 guidelines, adherence to polyp surveillance guidelines increased in our hospital. However, nearly half of the colonoscopies were performed unnecessarily due to non-adherence. Furthermore, our results demonstrate that the 2020 guidelines have decreased the need for follow-up.

## Introduction

Colorectal polyps are abnormal growths of cells that form on the inside of the colon or rectum. Some colorectal polyps have the potential to develop into colorectal cancer [1]. The incidence of colorectal cancer in the UK is 40,000 people per year [2]. The majority of colorectal cancers arise from colorectal polyps [3]. A majority of the polyps discovered during colonoscopy are removed. Follow-up after polypectomy is referred to as post-polypectomy surveillance colonoscopy. Polyps are followed up depending on their number, size, and histological grade. Guidelines exist to determine the appropriate follow-up.

Surveillance guidelines and adherence

In early 2020, the British Society of Gastroenterologists (BSG), the Association of Coloproctology of Great Britain and Ireland (ACPGBI), and Public Health England (PHE) published joint guidelines regarding post-polypectomy surveillance [4]. This replaced the previously widely-used guidelines by the BSG/ACPGBI published in 2002 [5] and updated in 2010 [6]. The 2002 guidelines and the 2010 update stratified colonoscopy findings into four categories: high, intermediate, low, and no risk. The category would determine the timing of follow-up. The 2020 guidelines were simplified into two categories: high and no-risk findings. The 2020 guidelines were the first to consider the role of the National Bowel Cancer Screening programme in detecting early cancers. Furthermore, it takes into account evidence to suggest that patients with low-risk polyps that are removed during initial colonoscopy have an equal chance of developing colorectal cancer as the general population [7].

Adherence to such guidelines is important, but previous research demonstrates poor adherence [8,9]. One systematic review and meta-analysis showed that over 50% of surveillance colonoscopies are performed too soon or too late [10].

Colonoscopies are unpleasant and expensive, and if unnecessary will delay the diagnosis and treatment of other patients who truly require investigation. Colonoscopies have small but significant risks associated with them. One study showed that the side effects of bleeding were 1:400, bowel perforation (needing surgery) 1:2,000, and death 1:30,000 [11]. Furthermore, the cost of each therapeutic colonoscopy is around £700 according to the NHS National cost schedule [12].

Aim

The aim of this study was to determine clinician adherence to the 2020 BSG/ACPGBI/PHE guidelines compared to the 2010 guidelines. Data were also examined to discover the number and cost of colonoscopies saved as a result of the 2020 guidelines.

This article was previously presented as a meeting poster at the 2022 Association of Surgeons of Great Britain and Ireland (ASGBI) Annual Conference on 3rd May 2022.

## Materials and methods

A retrospective review was undertaken of data collected from the Royal Devon University Healthcare NHS Foundation Trust colonoscopy database. The final 150 patients treated under the 2010 guidelines and the first 150 patients treated under the 2020 guidelines were selected. This sample size is large enough to detect an improvement in adherence from 40% to 60%, with just over 90% power at the two-sided 5% level of significance. The assumed percentage adherence under the 2010 guidelines of 40% is at the higher end of the plausible range, thus resulting in a conservatively large sample size requirement. Patients under the 2010 guidelines were collected over 6 months in 2019. Patients under the 2020 guidelines were collected over 8 months in 2020. Patients were selected if this was their first (baseline) colonoscopy. Each colonoscopy report was analysed by two authors of this study for findings and follow-up recommendations. A patient was deemed to be adherent if their colonoscopy report specified a follow-up time interval equal to that recommended by the guidelines. In total, 166 patients treated under the 2010 guidelines and 143 patients treated under the 2020 guidelines were selected from the colonoscopy database for analysis.

Patients were excluded from the analysis if: at baseline, colonoscopy malignancy or an alternative diagnosis was discovered; known familial colorectal cancer; patients underwent incomplete polypectomy or patients who declined follow-up. After exclusion criteria were applied, 152 and 133 patients treated under the 2010 and 2020 guidelines respectively were included in the study. In each study group, the percentage of patients for whom adherence was met was reported. The difference in adherence percentage was compared using a two-sample test of independent proportions using Stata statistical software. Costs were estimated using the NHS National Schedule of Costs. The unit price of therapeutic colonoscopy, 19 years and over the financial year 2019/20 was utilised, which was £702.71 per therapeutic colonoscopy [12].

## Results

**Figure 1:**
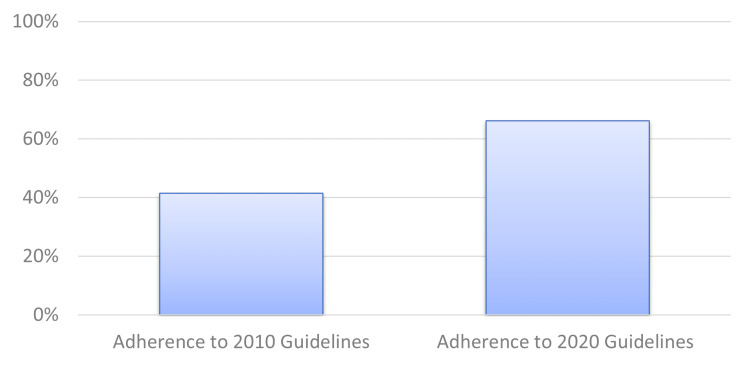
Clinician adherence to Post-polypectomy Surveillance Guidelines

Under the 2010 guidelines, 41.4% (63/152) of patients were adherent. Under the 2020 guidelines, 66.2% (88/133) of patients were adherent (Figure 1). There was a statistically significant increase in adherence of 24.7% (95% confidence interval 13.5% to 35.9%, p<0.0001). 

As seen in Table 1, under the 2010 guidelines, 62% (55/89) of non-adherent patients received surveillance colonoscopy sooner than recommended and 36% (32/89) of non-adherent patients underwent colonoscopy later than recommended. Under the 2020 guidelines, 36% (16/45) of non-adherent patients received surveillance colonoscopy sooner than recommended, and 62% (28/45) of patients received follow-up when it was not recommended at all. 

**Table 1:** The percentages and numbers of patients non-adherent to Post-polypectomy Surveillance Guidelines with a breakdown of reasons

Guidelines	Non-adherent patients	Reasons for non-adherence
2010 Guidelines (152 patients)	58.6% (89 patients) non-adherent	61.8% (55/89 patients) Colonoscopy sooner than recommended
		2.2% (2/89 patients) Colonoscopy not recommended
		36.0% (32/89 patients) Colonoscopy later than recommended
2020 Guidelines (133 patients)	33.8% (45 patients) non-adherent	35.6% (16/89 patients) Colonoscopy sooner than recommended
		62.2% (28/89 patients) Colonoscopy not recommended
		2.2% (1/89 patients) Colonoscopy later than recommended

Cost estimation

Approximately 37% (35/95) of patients who would have been followed up under the 2010 guidelines did not receive any follow-up due to the 2020 guidelines. This represents a cost saving of £24,594.85 in the study group, or £36,892.28 per year in our hospital.

Approximately 47% (28/60) of patients treated under the 2020 guidelines had surveillance colonoscopy planned when the guidelines recommended no follow-up. This represents an estimated unnecessary additional cost of £19,675.88 in the study group, or £29,513.82 per year in our hospital.

## Discussion

There was a marked increase in adherence to the 2020 BSG/ACPGBI/PHE guidelines compared to the 2010 guidelines. Unusually, for new guidelines, the 2020 update recommends less follow-up. As endoscopy units are under so much pressure, this may have been the driver for the observed greater adherence. Another reason might be that the 2020 guidelines are simpler to follow, given there are only two follow-up pathways rather than four pathways under the 2010 guidelines.

Previously published data shows adherence rates to polyp guidelines are poor, both in the UK and globally. One British district general hospital demonstrated that the 2002 BSG guidelines were successfully followed in only 17.7% of patients [13]. An Irish study of 363 polyp patients showed that 44.1% of patients were adherent to the 2002 guidelines [14]. In a European study of 2,997 patients, only 11% of patients were adherent to their respective Dutch guidelines [9]. Our data supports these findings.

The most common reason for non-adherence was that surveillance follow-up was planned earlier than recommended or planned when not recommended at all (Table 1). One possible explanation for this is clinician anxiety, the desire to follow up with their patients despite a negative result, or perceived patient anxiety that they might be concerned they have a disease despite the reassuring results of their baseline colonoscopy.

Performing unnecessary colonoscopies on patients who do not need them is unpleasant and potentially unsafe. Research shows post-colonoscopy bleeding rates were 1:400, bowel perforation (needing surgery) rates 1:2,000, and death rates 1:30,000 [11].

We estimated there would be 37% fewer patients followed up in our study group because of the 2020 guidelines compared to the 2010 guidelines with an estimated cost saving of £36,892.28 per year in our hospital department. However, 47% of patients treated under the 2020 guidelines had planned colonoscopies that were not recommended, costing an avoidable £19,675.88. Our data indicate that the 2020 guidelines offer a marked reduction in endoscopy demand for the surveillance of polyps. However, non-adherence still contributes to unnecessary costs and increases demand on endoscopy services. Increased adherence will likely mean patients who need their colonoscopy will receive it on time and more colorectal cancers will be identified at an earlier stage.

Approximately 15% of the 500,000 colonoscopies performed each year in the UK are for polyp surveillance [4]. If our data are extrapolated nationwide, then the 2020 guidelines could save more than 25,000 colonoscopies each year, the equivalent of £17,500,000.

Limitations

Patients were selected as the final 150 patients treated under the 2010 guidelines and the first 150 patients treated under the 2020 guidelines in our hospital. This study assumed once the 2020 guidelines were published, clinicians updated their clinical practice immediately. However, it often takes time and education for a full department to embrace new guidelines, and therefore, the true rate of adherence to the 2020 guidelines may be higher now than our data.

Cost estimation is made using national average costs published by the NHS national cost collection scheme. However, there remain additional costs of polyp surveillance which were not included in our estimation such as histology costs. We decided to use the cost of a ‘therapeutic colonoscopy’ because any colorectal polyps found on the colonoscopy should be removed. The precise costs are not known, as there are some factors that could increase the costs (for example multiple colonoscopies) and factors that could decrease costs (for example when no polyps are found).

Coronavirus Disease 2019 (COVID-19) and the resultant global pandemic occurred simultaneously with the introduction of the 2020 guidelines. It is likely that COVID-19 did alter the clinician adherence rate to the 2020 guidelines during the pandemic but to what extent is unclear.

## Conclusions

This retrospective study shows that clinician adherence to the 2020 BSG/ACPGBI/PHE guidelines has increased compared to the now-retired 2010 guidelines. However, it is clear that some patients continue to have an unnecessary follow-up that is unpleasant for them and costly to the National Health Service. Given that the 2020 guidelines require fewer patients to be followed up, it is paramount that clinicians adhere to them in order to avoid unnecessary colonoscopies. More work needs to be done to identify and address clinician barriers to adhering to polyp surveillance guidelines.
